# Studies on the Dermal and Ocular Irritation of Prodigiosin Isolated from *Zooshikella rubidus*

**DOI:** 10.5487/TR.2009.25.4.237

**Published:** 2009-12-30

**Authors:** Yong-Sook Kim, Jong-Myoung Choi, Jung-Hoon Yoon, Myung-Jin Choi, Md. Ahsanur Reza, Seung-Chun Park

**Affiliations:** 111 Present Address: Department of Fashion Design Information, Chungbuk National University, Cheongju, 361-763 Korea; 211 Korea Research Institute of Bioscience and Biotechnology 111 Gwahangno, Yuseong-gu, Daejeon, 305-806 Korea; 311 Laboratory of Veterimary Pharmacokinetics & Pharmacodynamics, College of Veterinary Medicine, Kyungpook National University, Daegu, 702-701 Korea

**Keywords:** *Zooshikella*, Prodigiosin, Eye irritation study, Skin irritation study

## Abstract

This study was carried out to investigate the irritation of the prodigiosin isolated from Zooshikella rubidus on the skin and eyes in New Zealand white rabbits. The tests were performed on the basis of Korea Food and Drug Administration (KFDA) guidelines. Prodigiosin induced severe eye irritation at high concentration (0.5 g/site/ml) but there was no eye irritation at low concentration (0.3 mg/site/ ml). The primary irritation index was calculated from higher concentration (0.5 g/site/ml) to lower concentration (0.3 mg/site/ml). There were found non-irritation or induced mild irritation at lower concentration of prodigiosin application. On the basis of this study, it could be concluded that the prodigiosin may be non-irritant to mild irritant of usual application at lower concentration (0.3 mg/site) resulting it is safe and useful in dyeing technology of fabrics.

## INTRODUCTION

Natural dyes are classified into plant, animal and mineral dye. Natural dyes are produced from natural materials like flower, tree, glass and bugs. In Old Stone Age, dye was started to use for fabrics. Many people suppose that dye was used to make juice to medical purpose at first time (Chung, 2002). Most of the dye are originated from vegetables like *persicaria tinctorium* , *Carthamus tinctorius* and *Schisandra chinensis Baillon*. But the vegetable dyes are limited on productivity, stability and reproducibility because of environmental effects.

Therefore, people need synthetic dye having mass production and storage capacity. It can contribute to development of processing and commercialization on fabrics (Yoo *et al*., 1998). But synthetic dyes induce water pollution and can make irritation or tumor in skin (Smith *et al*., 1991). Recently, natural dyes come into the spot-light with microbial dye (Li *et al*., 2008). Especially, microbiological dyes have a merit in good productivity and can make functional fabrics, though the using of microbiological dye is limited.

In this experiment, we isolated the prodigiosin from *Zooshikella rubidus,* is a novel bacterium producing the pigment and study the irritating effects of prodigiosin on the eye and skin of rabbits to ensure the safety for the using of microbial dye.

## METARIALS AND METHODS

***Test substance.*** *Zooshikella rubidus* was isolated on the basis of morphological, physiological features, chemical structure, 16S RNA sequence and molecular biological character and its named rubidus because of 'Ruby' like color. *Zooshikella rubidus* was obtained from the Biological Resource Center on 2008. 12 (No. KCTC 11448BP, Daejeon, Korea).

***Isolation of pigment.*** The stocked *Zooshikella rubidus* was transferred from Marine agar to Marine broth in 125 ml Erlenmeyer flask and incubated using rotary shaker at 120 rpm and transferred to 5 l Erlenmeyer flask (baffled type) containing 2.0 l broth. The supernatant and microbial cells were separated by centrifuga-

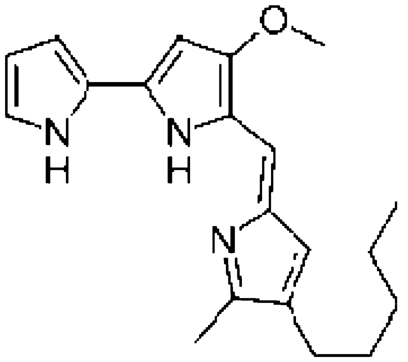

**Fig. 1.** A chemical structure of prodigiosin.

tion at 6,500 rpm for 15 minutes after two days incubation period. The same volume of ethanol was added with the microbial cells for extraction and the pigment was separated from the microbial solution by using rotator extractor. Finally pigment extract was concentrated at 50°C temperature. The purity of prodigiosin (Fig. 1) was determined at 99.68% in LC/MS (Fig. 2) by using of high performance liquid chromatography (HPLC).

***Animals.*** Male New Zealand White rabbits of 4 months of age were purchased from (ORIENT BIO INC., Korea) and acclimatized for 1 week. The animals were maintained on 12 hr light/dark cycle at 223°C temperature with humidity at 555%. The animals were housed in stainless steel cages, fed pellet diet and drink water ad libitum. This experiment was by Institute animal uses and care committee of Kyungpook National University (Approval number: VPT-BMPL-200705890000).

***Primary eye irritation test.*** The test procedure was done on the basis of the "Guidelines for Toxicity Studies of Drugs" provided by Korea Food and Drug Administration (KFDA) with slight modification (KFDA, 2009). Nine healthy rabbits were used for each group at high (0.5 g/site/ml) and low (0.3 mg/site/ml) concentration of prodigiosin treatment. Prodigiosin was infiltrated into conjuctival sac of the right eye and the left eye was served as a blank. The eyes of the 3 animals were used as rinsing group (washed with normal saline after 20~30 seconds of treatment) and the eyes of 6 animals were used as non-rinsing group. The eyes were examined and graded of ocular reaction at 1, 2, 3, 4, 7 days after treatment of prodigiosin. Besides, the corneal opacity, iris and conjunctival redness, edemas were recorded and classified according to guideline form of KFDA (Table 1).

***Dermal irritation test.*** The test procedure was done on the basis of the "Guidelines for safety test of the drugs" provided by KFDA (KFDA, 2009). Six healthy rabbits were used for each group at high (0.5 g/site/0.5 ml) and low (0.3 mg/site/0.5 ml) concentration of prodigiosin treatment. Each concentration of prodigiosin was administered by a moist cellulose patches onto shaved intact or abraded skin of rabbits. The patches were fixed for 24 h and the application sites were examined at 24 h and 72 hr after application and adjacent (left) areas of vehicle-treated skin of each rabbit served as control. The irritation score, primary irritation index (P.I.I.) and rating were recorded on the basis of the criteria described in Table 2 and examined whether the

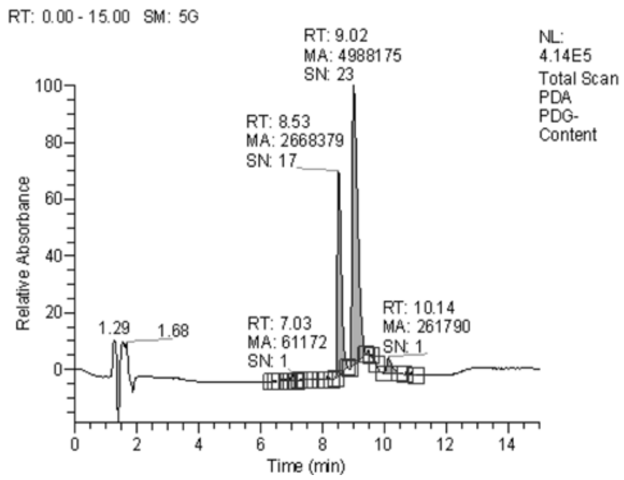

PEAK LIST
PDG-Content.RAW
RT: 0.00 - 15.00
Number of detected peaks: 12

| Apex RT | Start RT | End RT | Area | %Area |
|---|---|---|---|---|
| 6.42 | 6.36 | 6.5 | 5780.644 | 0.07 |
| 6.74 | 6.6 | 6.87 | 32116.84 | 0.39 |
| 7.03 | 6.9 | 7.19 | 61171.7 | 0.75 |
| 7.35 | 7.27 | 7.43 | 11509.12 | 0.14 |
| 7.54 | 7.45 | 7.64 | 10612.59 | 0.13 |
| 8.17 | 8.07 | 8.32 | 31311.75 | 0.38 |
| 8.53 | 8.44 | 8.82 | 2668379 | 32.58 |
| 9.02 | 8.9 | 9.41 | 4988175 | 60.9 |
| 9.49 | 9.43 | 9.56 | 31126.44 | 0.38 |
| 9.65 | 9.58 | 9.74 | 37480.78 | 0.46 |
| 10.14 | 9.99 | 10.49 | 261789.7 | 3.2 |
| 10.73 | 10.64 | 11.02 | 50718.97 | 0.62 |

**Fig. 2.** A purity of prodigiosin in high performance liquid chromatography. RT: retention time, MA: mean peak area, SN: signal-to-noise ratio.

**Table 1.** Irritation index on rabbit eyes after treatment of prodigiosin

| Rating | A.O.I | Evalution value M.O.I | Day-7 I.O.I |
|---|---|---|---|
| Non-irritant | 0-5 | 0 (after 48 hr) | |
| Minimal irritant | 6-15 | ≤ 5 (after 48 hr) | |
| Mild irritant | 16-30 | ≤ 5 (after 48 hr) | |
| Moderate irritant | 31-60 | ≤ 20 (after 7 days) | ≤ 30 (tested aniamals all) |
| Severe irritant | 61-80 | ≤ 40 (after 7 days) | ≤ 60 (tested animals all) |
| Extreme irritant | 81-110 | | |

M.O.I. (Mean ocular irritation index), total score/tested animal number in each observation time; A.O.I. (acute ocular irritation index), maximum among M.O.I. ; Day-7 I.O.I. (individual ocular irritation index), score of each animal on Day-7.

**Table 2.** Irritation rating for primary dermal reaction on rabbit skin after treatment of prodigiosin

| Ranging of P.I.I[a] | Rating |
|---|---|
| P.I.I. < 2 | Mild irritated |
| 2 ≤ P.I.I ≤ 5 | Moderate irritated |
| 5 < P.I.I. | Severe irritated |

[a]P.I.I.: Primary Irritation Index skin of rabbits had a capacity of cure by 11 days.

## RESULT AND DISCUSSION

Many people are interested in natural and microbiological dye in well-being life, although synthetic dye has a merit of productivity and stability for color. The prodigiosin is known as a metabolite which is isolated from *Eubacteriales* spp. and *Actinomycetales* spp (Robert, 1973). First it was isolated from *Serratia marcescens* (Robert, 1973 and Anta *et al*., 2006). In this study, the effects of prodigiosin from *Zooshikella rubidus* was investigated on eyes and skin of animals for the possibility of using in dye. Therefore, the experiment was carried out to study the irritation test with the treatment of prodigiosin on eyes and skin of rabbits through the KFDA guidelines to provide safety information (Shin *et al*., 1997; Lee *et al*., 2001).

The motality and clinical signs were not found of both groups of animals at high and low concentration during

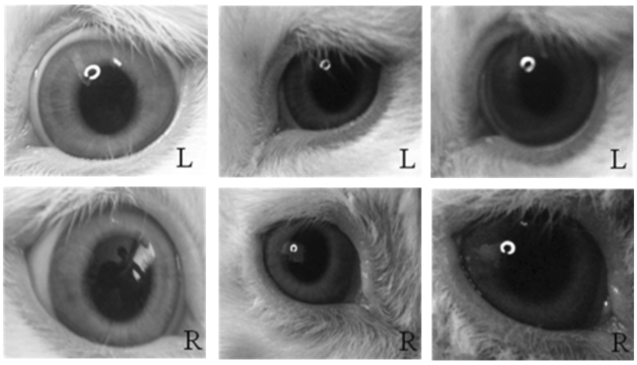

**Fig. 3.** Clinical signs in eyes of prodigiosin treated rabbits. Blank eye (L) and prodigiosin treated eye (R). Normal eye before treatment (A), No irritation sign at the period of 14 days after lower concentration of prdigiosin treatment (B) and moderate chemosis at the period of 14 days after higher concentration of prdigiosin treatment (C).

7 days of experiment in primary eye irritation test, but there was slight decrease in body weight at high concentration of prodigiosin application, but there were not observed significant differences. According to the M.O.I., A.O.I. and Day-7 I.O.I., the ocular clinical signs were classified. The result of ocular findings is shown in Table 3 and Fig. 3. The scores of the group of high concentration (0.5 g/site/ml) of prodigiosin treatment were 55 and 75 in ringing and non-ringing group respectively. M.O.I. score was decreased at 1, 2, 3, 4 and 7

**Table 3.** Ocular irritation reaction to prodigiosin in New zealand white Rabbits

| Test | Washing (Yes/No) | Mean ocular irritation index (M.O.I[a]) Observation time (day) | | | | | Acute ocular irritation index (A.O.I[b]) |
|---|---|---|---|---|---|---|---|
| | | 1 | 2 | 3 | 4 | 7 | |
| 0.5 g/site/ml | Y | 55 | 37 | 22 | 20 | 0 | 55 |
| | N | 72 | 75 | 73 | 43 | 23 | 75 |
| 0.3 mg/site/ml | Y | 0 | 0 | 0 | 0 | 0 | 0 |
| | N | 0 | 0 | 0 | 0 | 0 | 0 |

[a]M.O.I: (Mean ocular irritation index), total score/tested animal number in each observation time; [b]A.O.I: (acute ocular irritation index), maximum among M.O.I.

days successively and capacity of cure was observed by 11 days. A.O.I. score was 0 in the group at low concentration of prodigiosin application (0.3 mg/site/ml), so it means low concentration (0.3 mg/site/ml) can make safety in fabric dying.

In skin irritation test, the motality and clinical signs of both groups of animals were not observed at high and low concentration of prodigiosin treatment during 14 days of experiment. The body weight was decreased slightly till 4 days after high concentration of prodigiosin treatment, then the body weight was increased. This finding indicates that the high concentration of prodigiosin treatment can induce irritation but has capability of cure. The result of skin irritation on intact and abraded skin are shown in Table 4, 5 and Fig. 4. The high concentration of prodigiosin application induced severe

**Table 4.** Results of skin reactions treated with prodigiosin purified from *Zooshikellarubidus*

| Sites | | Control site | | | | | | | | 0.5 g/site (high dose) | | | | | | | |
|---|---|---|---|---|---|---|---|---|---|---|---|---|---|---|---|---|---|
| Change | | Erythema&eschar | | | | Edema | | | | Erythema&eschar | | | | Edema | | | |
| Phases | | Intact | | Abraded | | Intact | | Abraded | | Intact | | Abraded | | Intact | | Abraded | |
| Time (h) | | 24 | 72 | 24 | 72 | 24 | 72 | 24 | 72 | 24 | 72 | 24 | 72 | 24 | 72 | 24 | 72 |
| No. | Sex | | | | | | | | | | | | | | | | |
| 1 | male | 0 | 0 | 0 | 0 | 0 | 0 | 0 | 0 | 1 | 0 | 3 | 2 | 1 | 1 | 3 | 3 |
| 2 | male | 0 | 0 | 0 | 0 | 0 | 0 | 0 | 0 | 1 | 0 | 2 | 2 | 3 | 1 | 3 | 3 |
| 3 | male | 0 | 0 | 0 | 0 | 0 | 0 | 0 | 0 | 2 | 1 | 3 | 4 | 1 | 0 | 4 | 3 |
| 4 | male | 0 | 0 | 0 | 0 | 0 | 0 | 0 | 0 | 2 | 1 | 4 | 3 | 2 | 0 | 2 | 1 |
| 5 | male | 0 | 0 | 0 | 0 | 0 | 0 | 0 | 0 | 2 | 0 | 4 | 3 | 1 | 1 | 4 | 2 |
| 6 | male | 0 | 0 | 0 | 0 | 0 | 0 | 0 | 0 | 2 | 1 | 3 | 2 | 1 | 0 | 3 | 1 |
| Total score | | 0 | 0 | 0 | 0 | 0 | 0 | 0 | 0 | 10 | 3 | 19 | 16 | 9 | 3 | 19 | 13 |
| Mean score | | 0 | 0 | 0 | 0 | 0 | 0 | 0 | 0 | 1.7 | 0.5 | 3.2 | 2.7 | 1.5 | 0.5 | 3.2 | 2.2 |
| Sum of mean | | 0.0 | | | | | | | | 15.3 | | | | | | | |
| P.I.I[a] | | 0.0 | | | | | | | | 3.8 | | | | | | | |

Erythema was scored as follows: no erythema = 0, very slight erythema (barely perceptible) = 1, well-defined erythema = 2, moderate to severe erythema = 3, and severe erythema (beet redness) to slight eschar formation (injuries in depth) = 4. Edema formation was scored as follws: no edema = 0, very slight edema (barely perceptible) = 1, slight edema (edges of area well-defined by definite raising) = 2, moderate edema (raised approximately 1 mm) = 3, and severe edema (raised more than 1 mm and extending beyond area of exposure) = 4. [a]Primary irritation index = total score/4.

**Table 5.** Results of skin reactions treated with prodigiosin purified from *Zooshikella rubidus*

| Sites | | Control site | | | | | | | | 0.3 mg/site (low dose) | | | | | | | |
|---|---|---|---|---|---|---|---|---|---|---|---|---|---|---|---|---|---|
| Change | | Erythema&eschar | | | | Edema | | | | Erythema&eschar | | | | Edema | | | |
| Phases | | Intact | | Abraded | | Intact | | Abraded | | Intact | | Abraded | | Intact | | Abraded | |
| Time (h) | | 24 | 72 | 24 | 72 | 24 | 72 | 24 | 72 | 24 | 72 | 24 | 72 | 24 | 72 | 24 | 72 |
| No. | Sex | | | | | | | | | | | | | | | | |
| 1 | male | 0 | 0 | 0 | 0 | 0 | 0 | 0 | 0 | 1 | 0 | 1 | 0 | 0 | 0 | 0 | 0 |
| 2 | male | 0 | 0 | 0 | 0 | 0 | 0 | 0 | 0 | 1 | 0 | 1 | 0 | 0 | 0 | 0 | 0 |
| 3 | male | 0 | 0 | 0 | 0 | 0 | 0 | 0 | 0 | 1 | 0 | 0 | 0 | 0 | 0 | 1 | 1 |
| 4 | male | 0 | 0 | 0 | 0 | 0 | 0 | 0 | 0 | 1 | 0 | 0 | 0 | 0 | 0 | 0 | 0 |
| 5 | male | 0 | 0 | 0 | 0 | 0 | 0 | 0 | 0 | 1 | 0 | 0 | 0 | 0 | 0 | 1 | 0 |
| 6 | male | 0 | 0 | 0 | 0 | 0 | 0 | 0 | 0 | 1 | 0 | 0 | 0 | 0 | 0 | 0 | 1 |
| Total score | | 0 | 0 | 0 | 0 | 0 | 0 | 0 | 0 | 6 | 0 | 2 | 0 | 0 | 0 | 2 | 2 |
| Mean score | | 0 | 0 | 0 | 0 | 0 | 0 | 0 | 0 | 1.0 | 0.0 | 0.3 | 0.0 | 0.0 | 0.0 | 0.3 | 0.3 |
| Sum of mean | | 0.0 | | | | | | | | 2.0 | | | | | | | |
| P.I.I[a] | | 0.0 | | | | | | | | 0.5 | | | | | | | |

Erythema was scored as follows: no erythema = 0, very slight erythema (barely perceptible) = 1, well-defined erythema = 2, moderate to severe erythema = 3, and severe erythema (beet redness) to slight eschar formation (injuries in depth) = 4. Edema formation was scored as follws: no edema = 0, very slight edema (barely perceptible) = 1, slight edema (edges of area well-defined by definite raising) = 2, moderate edema (raised approximately 1 mm) = 3, and severe edema (raised more than 1 mm and extending beyond area of exposure) = 4. [a]Primary irritation index = total score/4.

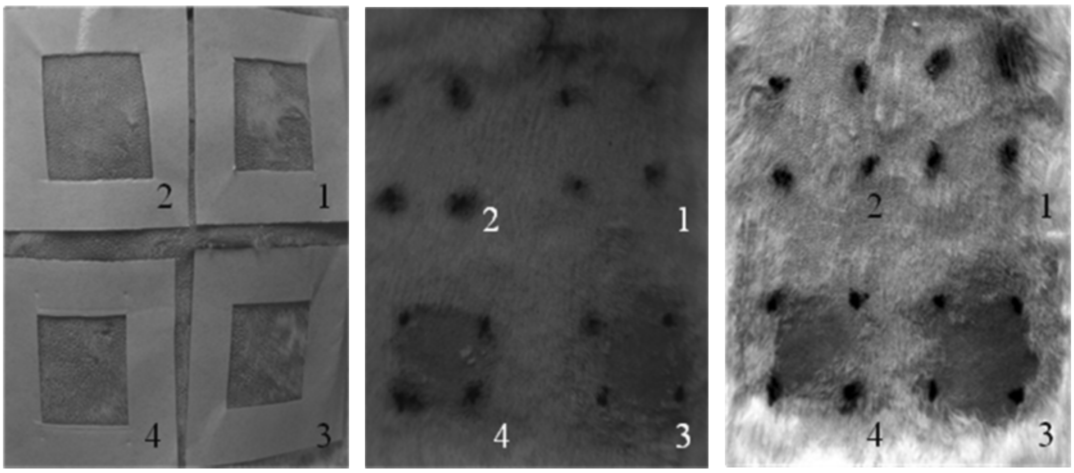

**Fig. 4.** Clinical signs in skin of prodigiosin treated rabbits. 1 and 2 regions were treated normal saline, but 3 and 4 regions were treated prodigiosin. And 1 and 3 were abraded skin. There was normal skin before prodigiosin application (A), mild irritation (3) and no irritation (4) sign in abraded skin at the period of 14 days after low concentration of prodigiosin treatment (B). But, there was erythema and edema (3) in abraded skin at the period of 14 days after high concentration of prodigiosin treatment (C).

erythema and eschar and P.I.I. score were 3.8 and 0.5 at higher concentration (0.5 g/site/0.5 ml) and lower concentration (0.3 mg/site/0.5 ml) of prodigiosin treatment respectively, resulting low concentration of prodigiosin application was found non-irritant or induced mild irritation. The severe erythema and mild eschar were observed with high concentration (0.5 g/site/0.5 ml) of prodigiosin application in abraded skin than intact skin. Therefore it can be concluded that prodigiosin is safe at less than 0.3 mg/ml as usual concentration.

Among many of the synthetic dyes, Disperse Black and Disperse use for the making polyester by 11% and 31%, induce severe skin irritation and eye irritation (Andrea *et al.*, 1998). The result is not compared due to lack of available literatures of the effect of natural dyes (prodigiosin ) on skin or eye irritation, although there are many information about toxicity and irritation of synthetic dyes (Ravindra *et al.*, 1999). But, it was reported that alizarin and purpurin derived from plant name for madder are associated with kidney damage and genotoxic effect (Ismene *et al.*, 2006). Compared with madder, it is known the low concentration (0.3 mg/ml) of prodigiosin is safe to use in dyeing of fabrics. For this reason, prodigiosin can be used in dyeing industries at low concentration (0.3 mg/ml) for its non-toxicity, high productivity and stability. On the other hand, prodigiosin is known for anti-inflammation, anti-bacteria, anti-malaria (Demain, 1995), and anti-tumor activity (Roser *et al.*, 2007). So, we expect that prodigiosin may make a novel functional role in dye of fabric technology of prodiginine like prodigiosin.

## ACKNOWLEDGMENTS

This work was supported by the Korea Research Foundation grant by the Korean government (KRF-2008-531-H00004).
